# Lateralizing value and clinicoradiological features of asymmetric last clonic jerks in temporal and extratemporal epilepsy

**DOI:** 10.1038/s41598-024-61401-y

**Published:** 2024-05-21

**Authors:** Furkan Saridas, Gizem Mesut, Yasemin Dinc, Aylin Bican Demir, Ibrahim Bora

**Affiliations:** Department of Neurology, Bursa Uludağ University Medicine Faculty, Bursa, Türkiye

**Keywords:** Epilepsy, Epilepsy

## Abstract

Seizure semiology and electroencephalograph (EEG) are very important for determining seizure type, hemisphere lateralization, or localization. Clinical symptoms of focal seizures, as well as findings at the onset or end of a focal to bilateral tonic–clonic seizure (FBTCS), are highly informative for lateralization. This study aimed to investigate the relationship of asymmetric last clonic jerk in patients with temporal or extratemporal lobe epilepsy with pathologies, localization, lateralization, or other semiological findings detected in neuroimaging or neuro psychometric tests and its positive predictive value for the detection of hemisphere lateralization based on seizure onset ictal EEG activation. 44 patients with asymmetric last clonic jerks (aLCJ) who were followed up in our VEM unit were randomized 1:1 with epilepsy patients without. In patients with ipsilateral automatism and contralateral posture or gustatory and olfactory hallucinations aLCJ was less or absent. In patients with unilateral tonic activity, aLCJ was more common. The positive predictive value of aLCJ for ictal EEG activation lateralization was 86.36%. In conclusion, asymmetric last clonic beat is valuable for lateralization of FBTCS and should be considered. Its presence strongly and reliably lateralizes to the side of seizure onset.

## Introduction

Seizure semiology and electroencephalograph(EEG) are very important for determining seizure type, hemisphere lateralization, or localization. VEM provides a detailed analysis of the semiological characteristics of seizures correlated with simultaneous EEG activity. Especially in temporal and extratemporal epilepsies, observation of seizures with VEM includes essential information ^1,2^. Routinely, seizure semiology is analyzed with EEG, neuropsychometric testing (NPT), and neuroimaging. Clinical symptoms in focal seizures, as well as findings at the onset or end of a FBTCS, are highly informative for lateralization ^3–5^. However, it is not possible to examine every patient with VEM, or pathology may not be detected in the recordings. Semiologic findings after the seizure onset are more valuable for these patients than those at the onset, as they can be more clearly seen by both the patient's companions and health professionals.

In temporal or frontal lobe epilepsies, the focal onset and asymmetric termination of the bilateral tonic–clonic phase of secondary generalized tonic–clonic seizures reliably lateralize the side of seizure onset. The asymmetric last clonic jerk occurs ipsilateral to the side of the seizure onset site ^6,7^. This study aimed to investigate the relationship of asymmetric last clonic jerk in patients with temporal or extratemporal lobe epilepsy with pathologies, localization, lateralization, or other semiological findings detected in neuroimaging or neuro psychometric tests and its positive predictive value for the detection of hemisphere lateralization based on seizure onset ictal EEG activation.

## Materials and methods

### Patient selection

**Figure 1:**
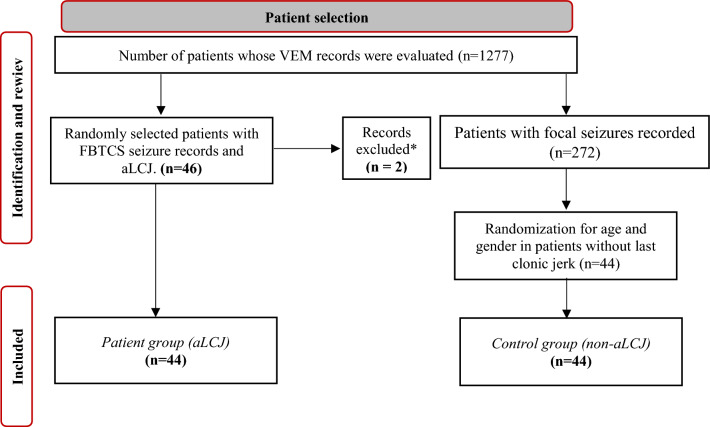
Patient selection flowchart. *In two patients, seizures with aLCJ were found to be psychogenic non-epileptic seizures other than FBTCS and were excluded from the study.

The records of the patients with FBTCS among the patients with focal seizures during hospitalization in the VEM unit of our center between 2005 and 2023 were retrospectively evaluated. Among them, 46 patients with asymmetric last clonic jerks were randomly selected. Two patients were also found to have seizures without electrophysiological correlates (psychogenic non-epileptic seizures) accompanied by last clonic jerks, and 44 patients were included in the study after exclusion (aLCJ). aLCJ patients were randomised in a 1:1 ratio according to age and sex by computer-assisted age and gender with other patients with focal seizures without last clonic jerks (control group; non-aLCJ) from the hospital database. (Fig. 1) (Informed consent was obtained from all subjects and/or their legal guardian(s), and all methods were performed in accordance with the relevant guidelines and regulations.)

### VEM protocol

VEM recordings and a continuous video recording were made using scalp electrodes in accordance with the international 10–20 electrode mounting system, with the data using 16–32-channel reference longitudinal and transverse bipolar montages. Spikes, sharp waves, spike and wave complexes temporally intermittent rhythmic delta activity, and continuous focal slow wave activity (theta or delta) were determined. Electrodes with rhythmic theta or delta waves, spike waves or sharp waves before ictal activity were considered as ‘localization of the initial ictal activity’. Anti-seizure medications were reduced or discontinued to facilitate the onset of seizures. After adequate seizure recording, appropriate anti-seizure medication was continued at effective doses. A total of 102 VEM recordings with scalp electrodes (58 patient groups; 5642 h and 44 control group; 3816 h for a total of 9458 h), number of seizures observed, semiologic findings, ictal and interictal EEG findings were retrospectively evaluated. Four hundred twenty-eight seizures (201 and 227, respectively) were analyzed during the recording period.

### Other assessments

In addition, all patients underwent NPT during hospitalization. High-resolution 3 Tesla (3 T) Magnetic Resonance Imaging (MRI) was available in all patients. 18F-fluorodeoxyglucose Positron Emission Tomography (FDG PET) results were recorded for patients (56 patients) who underwent 18F-fluorodeoxyglucose Positron Emission Tomography (FDG PET) at the time of hospitalization or within one year before hospitalization, where possible. NPTs were administered by two clinical neuropsychologists experienced in epilepsy. Visual-spatial (occipital regions to anterior pole of temporal lobe, posterior parietal cortex, frontal eye fields, and dorsal visual system), language (classical speech zones, typically in the left dominant hemisphere, including Wernicke's and Broca's areas, and the angular gyrus), attention (ascending reticular activating system, superior colliculus, Five different modalities including thalamus, parietal lobe, anterior cingulate cortex, and the frontal lobe), memory (hippocampal-entorhinal cortex complex, frontal regions, left parietal cortex), and executive functions (frontal-subcortical circuits, including dorsolateral prefrontal, orbital frontal, and anterior) were evaluated in each patient. According to the results, pathological localization and lateralization were categorized as frontal, temporal, right, left, and bilateral.

Reports of all FDG PET scans and MRIs by neuroradiologists, EEG recordings and NPT results were evaluated by a neurologist and two experienced blinded epileptologists, one of whom was a clinical neurophysiologist. After evaluation by more than one expert, the findings were finalized by consensus and analyzed in comparison with clinical characteristics.

### Statistical analysis

Descriptive statistics are presented as frequency and percentage for qualitative data. Pearson Chi-square, Fisher-Freeman-Halton, and Fisher's Exact Chi-square tests were used to analyze categorical data. Kappa coefficient and McNemar test were calculated to evaluate the agreement of qualitative data. The significance level was set as α = 0.05. Statistical data analysis was performed using IBM SPSS 28.0 (IBM Corp. Released 2021. IBM SPSS Statistics for Windows, Version 28.0. Armonk, NY: IBM Corp.) statistical package program.

### Ethics approval

Ethical permission had been obtained. (Bursa Uludağ University Faculty of Medicine Clinical Research Ethics Committee, Approval no: 2023-24/37).

## Results

A total of 44 patients, 19 females, and 25 males, had a median age of 35 (18–53) years during video EEG monitoring. The mean duration of VEM recording was 107.47 ± 47.79 h (patient group 128.22 ± 58.43, control group 86.72 ± 18.09 h). Six patients were on monotherapy, 24 were on dual, and 14 were on three or more anti-seizure medications (12, 19, and 13 in the control group, respectively). Epilepsy syndromes and anti-seizure medications of the patients are given in Table 1. A total of 88 MRIs (44, 44, respectively), 60 FDG PET scans (35 in 31 patients and 25 in 25 patients, respectively), and 82 NPTs (42, 40, respectively (two and four patients did not comply with the tests) were evaluated. The dominant hand (right) was 39 and 41, respectively.

**Table 1:** Descriptive findings of clinical, electrophysiological and imaging studies.

	aLCJ	non-aLJC
Age^a^	35 (18–53)	
Sex^a^ (Female n, %)	19 (43.2%)	
Epilepsy Type (frontal, temporal, other)	22, 21, 1	11, 33, –
History of Epilepsy Surgery	4 (9%)	2 (4.5%)
Normal MRI	18 (40.9%)	15 (34%)
Same lateralisation between MR focus and ictal EEG focus^b^	9 (60%)	15 (75%)
Follow-up period	110 (24–290)	96 (24–96)
Number of seizures analysed	4 (1–33)	3 (1–51)
PET	31 (70.4%)	25 (56.8%)
Same lateralisation between PET focus and ictal EEG focus^b^	12 (75%)	7 (53.8%)
Anti-seizure medication		
Levetiracetam	31	37
Carbamazepine	22	17
Valproate	13	11
Lamotrigine	10	4
Oxcarbazepine	9	3
Phenytoin	6	0
Topiramate	5	1
Lacosamide	2	9
Vigabatrin	1	0
Gabapentin	1	0
Phenobarbital	0	1
Zonisamide	0	4
Clobazam	0	3
Pregabalin	0	1

Descriptive data are given as n (%) or median (min–max).

^a^The control group (non-aLCJ) was randomised 1:1 for age and gender.

^b^Except for bilateral/bifocal foci and patients with diffuse/spread lesions on MRI.

**Figure 2:**
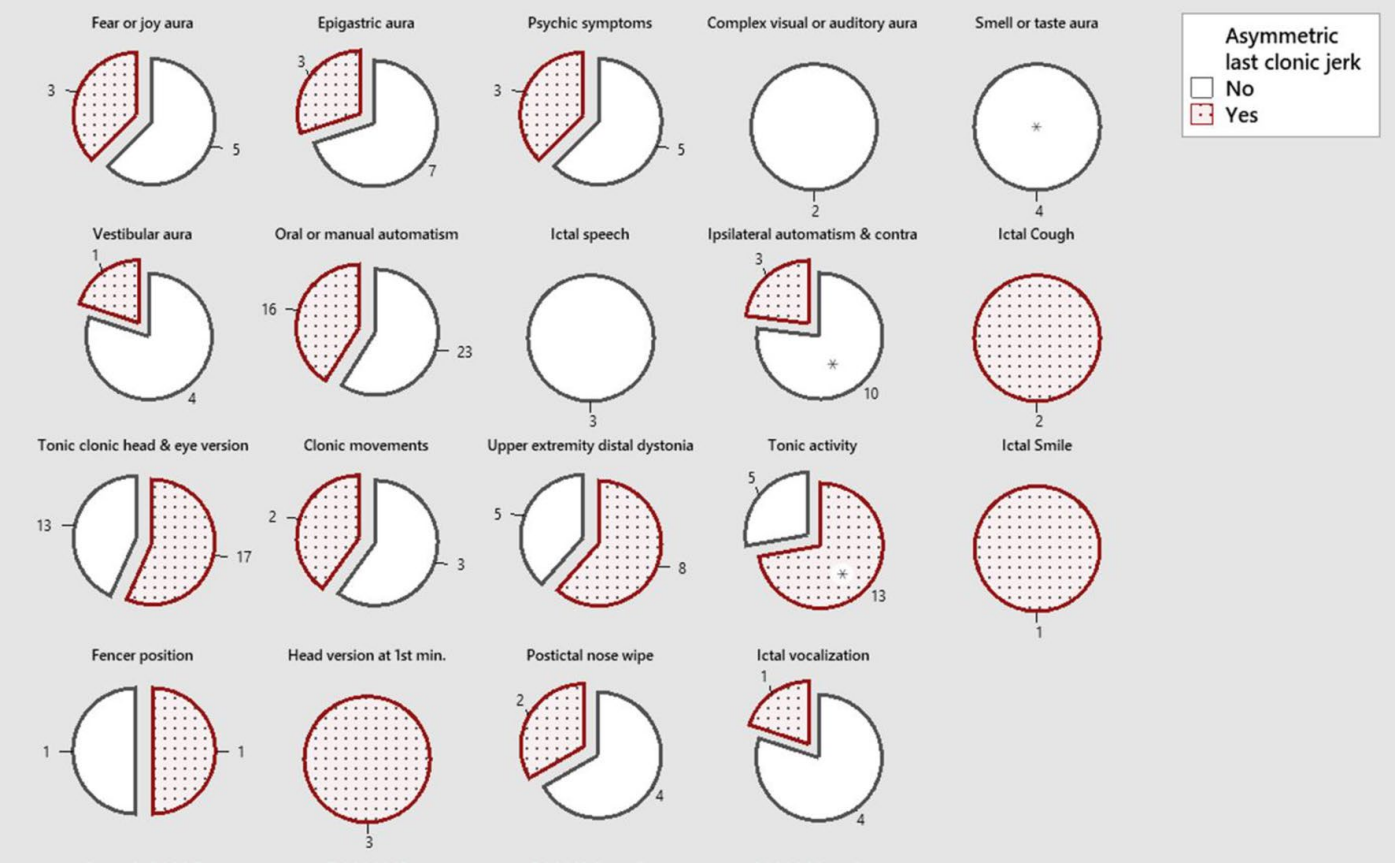
Frequency of accompaniment of other semiological findings. Ipsilateral automatism and contralateral posture p = 0.035, Olfactory or taste aura (Gustatory and olfactory hallucinations) p = 0.041, Tonic activity (Unilateral) p = 0.034, respectively.

**Figure 3:**
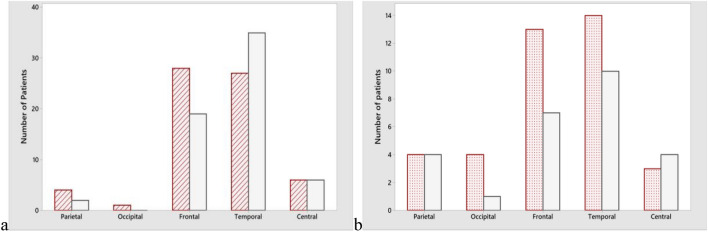
Electrophysiological localization differences of the initial ictal activity in patients with aLCJ compared to those without. (**a**) Ictal rhythmic teta/delta activity. (**b**) Ictal spike or sharp wave, (p > 0.05). Red; aLCJ, grey; non-aLCJ.

There was no difference for the dominant hand in patients with aLCJ. In patients with ipsilateral automatism and contralateral posture or gustatory and olfactory hallucinations, which are common in temporal lobe epilepsy among other semiologic findings, aLCJ was less or absent. In patients with unilateral tonic activity, aLCJ was more common. (Fig. 2, appendix-1) There was no difference in other semiologic findings. There was no difference in pathologies detected on MRI. (Table 2) aLCJ was slightly more frequent for pathology localization in patients with diffuse/spread or parietal region lesions on MRI. There was no localization difference for PET. For NPT, aLCJ was more frequent in patients with dysfunction in the frontal region than in patients with dysfunction in the temporal region. (Table 3) In the electrophysiological recording, interictal occipital region sharp or spike-wave activation was observed in patients with aLCJ (p = 0.021), while temporal region interictal rhythmic teta or delta wave activation was less frequent (p = 0.017). There was no difference for localization of the initial ictal activity. (Fig. 3, appendix-2) MRI, PET, and NPT ictal EEG were statistically significantly associated with baseline hemispheric activity lateralization. For patients with bilateral or bifocal activation at baseline, the results obtained only in NPT were significant for bilateral involvement. In a total of 40 patients (90.9%) with 18 right and 22 left asymmetric aLCJ, the positive predictive value of aLCJ for ictal EEG activation lateralization was 86.36% (AUC: 0.758, p = 0.002), mostly concurrence (κ = 0.485).

**Table 2:** Comparison of MR pathology in patients with and without aLCJ.

Pathology	n	aLCJ (%)	non-aLCJ (%)	P value
Normal	33	18 (40.9)	15 (34.1)	0.509
Non-specific ischemic gliosis	10	7 (15.9)	3 (6.8)	0.179
Mesial temporal sclerosis	18	6 (13.6)	12 (27.3)	0.113
Atrophy	11	6 (13.6)	5 (11.4)	0.747
Encephalomalacia	3	3 (6.8)	–	0.078
Cystic lesion	6	3 (6.8)	3 (6.8)	1
Ulegri	3	2 (4.5)	1 (2.3)	0.557
Temporal dysplasia	3	1 (2.3)	2 (4.5)	0.557
Undiagnosed hyperdense lesion	4	1 (2.3)	3 (6.8)	0.306
Polymicrogyria	1	1 (2.3)	–	0.315
Cortical dysplasia	4	1 (2.3)	3 (6.8)	0.315
Av-fistula	1	–	1 (2.3)	0.315
Cavernoma	1	–	1 (2.3)	0.315
Demyelinating plaque	1	–	1 (2.3)	0.315
Cerebellopontin angle tumor	1	–	1 (2.3)	0.315
Residual signs of hypoxic ischemic encephalopathy	1	–	1 (2.3)	0.315

**Table 3:** The relationship between pathologies detected in patients with aLCJ and localization.

Localization comparison		n	aLCJ (%)	non-aLCJ (%)	P value
MRI	Diffuse/spread	7	6 (13.6)	1 (2.3)	**0.049**
	Paragagittal	1	1 (2.3)	–	0.315
	Frontal	15	7 (15.9)	8 (18.2)	0.777
	Lateral temporal	12	5 (11.4)	7 (15.9)	0.534
	Mesial temporal	24	10 (22.7)	14 (31.8)	0.338
	Parietal	7	6 (13.6)	1 (2.3)	**0.049**
	Occipital	5	3 (6.8)	2 (4.5)	0.645
	Cerebellum	2	–	2 (4.5)	0.153
	Cerebellopontine	1	–	1 (2.3)	0.315
	External capsule	1	1 (2.3)	–	0.315
PET	Mesial temporal cortex	26	11 (36.7)	15 (60)	0.084
	Lateral temporal cortex	12	6 (20)	6 (24)	0.661
	Insular cortex	4	4 (13.3)	–	0.058
	Temporal anteromedial	9	3 (10)	6 (24)	0.162
	Frontal cortex	3	1 (3.3)	2 8)	0.448
	Precentral gyrus	1	1 (3.3)	–	0.357
	Parieto-occipital	4	4 (13.3)	–	0.058
	Central sulcus	1	1 (3.3)	–	0.357
	Parietal	2	–	2 (8)	0.269
	Basal ganglia	1	–	1 (4)	0.269
	Thalamus	1	–	1 (4)	0.269
NPT	Frontal	30	20 (47.6)	10 (25)	**0.034**
	Temporal	32	11 (26.2)	21 (52.5)	**0.015**

Significant values are in [bold].

## Discussion

The diagnosis of epileptic seizures depends on recognizing the behavioral characteristics of these episodes. The semiology of seizures in epilepsy forms the basis of clinical description. Some semiological features provide essential clues for hemisphere lateralization and lobar localization. However, this alone may not be sufficient; it may be misleading in multifocal epilepsy, or sometimes, it may be a finding of seizure spread rather than seizure onset location. In determining seizure type and localization of onset, EEG and semiology are also very valuable. VEM allows detailed analysis of the semiological features of seizures correlated with simultaneous EEG activity. Especially in temporal and extratemporal epilepsies, observation of seizures with VEM provides essential information for lateralization and localization. However, it is not possible to examine every patient with VEM.

Temporal lobe seizures are the most common site of focal seizures. Usually, aura and psychic symptoms are followed by motor activity. Frontal lobe epilepsies are the most common extratemporal seizures. They usually have sudden onset, short duration, a very short postictal confusion period, and seizures are generally clustered. Different semiologic features are seen according to the onset or location of the seizure. These features may be more frequent or specific in temporal or frontal lobe seizures and have localization or lateralization value. For example, in temporal lobe epilepsy, where auras are more common, elevated epigastric sensation, Fear or joy, déjà, and jamais vu, visceral or auditory illusions, complex auditory or visual hallucinations, and taste or olfactory hallucinations are seen. However, although these auras are usually of localizing value, they do not have lateralizing significance. Findings such as oral or manual automatism, psychic symptoms, ipsilateral automatism and contralateral posture, postictal cough, or ictal speech arrest or preservation during seizures indicate lateralization to the non-dominant hemisphere or localization to the ipsilateral or contralateral temporal lobe. For frontal seizures, tonic–clonic head and eye version, unilateral clonic movements of the face or one of the upper or lower extremities, a slow, involuntary abnormal posture with a rotatory component distal to the upper extremities, unilateral tonic activity, unilateral smile, and four or fencing indicating contralateral lateralization, short-term (2 s) and greater than 30° head version in the first minute of the seizure, unilateral hand automatisms, postictal nose wiping after seizure termination, unilateral blinking at the onset of the seizure, ictal vocalization and asymmetric termination of the clonic phase at the end of the FBTCS evaluated in this article indicate ipsilateral lateralization. (seizure onset hemisphere and long clonic phase are ipsilateral) ^7–10^. Since incorrect seizure classification may lead to inappropriate treatment selection or unnecessary investigations, the correct seizure classification should be made first; then, the lateralization value should be interpreted ^11^.

In our study, the association of aLCJ with ipsilateral automatism and contralateral posture, smell or taste aura, which are more common in temporal lobe epilepsy, and unilateral tonic activity, which is more common in frontal lobe epilepsy, was significant. There was no significant association of aLCJ with other semiologic features. The mechanisms leading to aLCJ need to be clarified and speculative. In focal seizures, it is thought that earlier active inhibition in the hemisphere of seizure onset or earlier depletion in the retained hemisphere may cause this phenomenon ^12,13^. In our study, there was a significant association of aLCJ with ipsilateral automatism and contralateral posturing or gustatory and olfactory hallucinations, which are common in temporal lobe epilepsies, especially in mesial temporal lobe epilepsy (most commonly sclerosis). This association may be related to abnormally organized neuronal network structures based on the sclerosis of mesial temporal structures over a long period. On the other hand, the significant association we found with unilateral tonic posture, which is more common in frontal lobe epilepsies, may be related to the proximity to the ictal onset localization. However, since there is no difference in the frequency of association with many other semiologic findings, the pathogenesis remains to be elucidated.

Literature studies evaluating the relationship between semiological features and organic pathologies are limited. Our study did not find a significant difference between aLCJ and the MRI pathologies detected in our cohort. However, diffuse/spread or parietal localization on MRI was mildly associated with aLCJ, although we did not find significance for localization on FDG PET. This suggests that diffuse/spread lesions may lead to more depletion in the previously involved hemisphere or that lesions in the parietal region, which are located right next to the seizure onset localization in patients with frontal lobe epilepsy, may be associated with aLCJ by undergoing earlier inhibition.

Notably, interictal temporal slow wave activation is less common in those with aLCJ, and the difference between temporal and frontal localizations for interictal or ictal activation is reduced in favor of frontal. This suggests that aLCJ may be more frequent in frontal lobe epilepsy. The higher frequency of aLCJ in patients with initial activation in the occipital region may be related to the faster spread of occipital lobe epilepsies compared to temporal lobe epilepsies and earlier hemispheric exhaustion ^14^. However, the small number of patients makes confirmation difficult.

Although advanced invasive examinations and imaging studies have improved considerably, VEM, ictal semiologic findings, and analysis of ictal discharges are still important in epileptic focus detection ^15^. Asymmetric motor signs in the semiology of FBTCS, such as forced head version, fencing, and aLCJ, have remarkable accuracy for seizure origin lateralization. aLCJ occurs ipsilaterally next to the seizure onset site, and FBTCS of frontal or temporal origin have a high positive predictive value for lateralization ^6,7,12^. On the other hand, it should be kept in mind that aLCJ can be seen not only in focal onset seizures but also in primary generalized tonic–clonic seizures and even in psychogenic non-epileptic seizures (PNES) (aLCJ was detected in two patients with PNES who were excluded from our study).^16^ However, epilepsy surgery are currently the gold standard for the confirmation of lateralisation findings. In our cohort, the number of patients with aLCJ who underwent epilepsy surgery during follow-up was too small to be included in the statistical evaluation (n = 8). Therefore, unfortunately, we could not perform this analysis in this study. Nevertheless, the positive predictive value of asymmetric last clonic jerks for ipsilateral lateralisation was 86.36% when evaluated with ictal EEG activation. This result confirms that it is a robust semiological finding for ipsilateral lateralisation. In our study, the positive predictive value of the asymmetric last clonic jerk for ipsilateral lateralization was 86.36%. This result confirms that it is a robust semiological finding for ipsilateral lateralization.

## Conclusion

In patients with refractory epilepsy, seizure semiology is very important in combination with neuroimaging and neuropsychometric tests for accurate seizure classification, appropriate anti-seizure medication or appropriate treatment modality. VEM may not be suitable for evaluating all patients, or data may not be obtained during hospitalization. In this context, the asymmetric last clonic beat is precious for lateralization of FBTCS and should be considered. Its presence strongly lateralizes to the side of seizure onset.

### Study limitation

In this study, we did not perform a retrospective evaluation for poor/good surgical outcomes because the proportion of patients with asymmetric last clonic jerk who underwent epilepsy surgery was small. The main limitations of the study are the small number of patients, the fact that the findings were obtained from EEG examinations with superficial electrodes and were not confirmed by surgery, which has recently been the gold standard. Especially in patients with rapid generalization, muscle artifacts may be confusing as they may obscure the onset and propagation pattern. However, despite this, the PPV was found to be high, indicating that the result is robust.

### Supplementary Information

Supplementary Information.

## Data Availability

The datasets used and/or analysed during the current study available from the corresponding author on reasonable request.
