# Supplementary material for: Lateralizing value and clinicoradiological features of asymmetric last clonic jerks in temporal and extratemporal epilepsy
Source: Sci Rep. 2024 May 21;14:11578. doi: 10.1038/s41598-024-61401-y (PMC11109186; doi:10.1038/s41598-024-61401-y)
Supplement: Supplementary file 1 — Supplementary Information. [file 41598_2024_61401_MOESM1_ESM.docx]

**Appendix**

**Appendix-1:** Occurrence rates and association relationships of other semiological findings in patients with aLCJ.

|  | **Features** | **n** | **aLCJ (%)** | **non-aLCJ (%)** | **P value** |
| --- | --- | --- | --- | --- | --- |
| **Dominant hand** | Right | 80 | 39 (88.6) | 41 (93.2) | 0.458 |
| **Temporal lobe epilepsy semiology** | Oral or manual automatism | 39 | 16 (36.4) | 23 (52.3) | 0.133 |
|  | Fear or joy | 8 | 3 (6.8) | 5 (11.4) | 0.458 |
|  | Rising epigastric sensation | 10 | 3 (6.8) | 7 (15.9) | 0.179 |
|  | Psychic symptoms | 8 | 3 (6.8) | 5 (11.4) | 0.458 |
|  | Ipsilateral automatism & contralateral posture | 13 | 3 (6.8) | 10 (22.7) | **0.035** |
|  | Postictal cough | 2 | 2 (4.5) | - | 0.153 |
|  | Vestibular aura | 5 | 1 (2.3) | 4 (9.1) | 0.167 |
|  | Ictal speech arrest or preservation | 3 | - | 3 (6.8) | 0.078 |
|  | Complex visual or auditory hallucinations | 2 | - | 2 (4.5) | 0.153 |
|  | Gustatory and olfactory hallucinations | 4 | - | 4 (9.1) | **0.041** |
| **Frontal lobe epilepsy semiology (contralateral)** | Tonic clonic head and eye version | 30 | 17 (38.6) | 13 (29.5) | 0.368 |
|  | Unilateral tonic posturing | 18 | 13 (29.5) | 5 (11.4) | **0.034** |
|  | Upper extremity distal dystonia | 13 | 8 (18.2) | 5 (11.4) | 0.367 |
|  | Unilateral clonic movements | 5 | 2 (4.5) | 3 (6.8) | 0.645 |
|  | Unilateral smile | 1 | 1 (2.3) | - | 0.315 |
|  | Fencing position | 2 | 1 (2.3) | 1 (2.3) | 1 |
| **Frontal lobe epilepsy semiology (ipsilateral)** | Unilateral hand automatism | 22 | 14 | 8 (18.2) | 0.140 |
|  | Head version at first minute of seizure | 3 | 3 (6.8) | - | 0.078 |
|  | Postictal nose wipe | 6 | 2 (4.5) | 4 (9.1) | 0.398 |
|  | Ictal vocalization | 5 | 1 (2.3) | 4 (9.1) | 0.167 |

**Appendix-2:** Differences in ictal and interictal localization findings in patients with aLCJ.

| **Eeg**  **Activity** | | **Localization** | **n** | **aLCJ (%)** | **non-aLCJ (%)** | **P value** |
| --- | --- | --- | --- | --- | --- | --- |
| **Interıctal EEG** | **Theta or delta wave activation** | **Parietal** | 14 | 10 (22.7) | 4 (9.1) | 0.080 |
|  |  | **Occipital** | 11 | 8 (18.2) | 3 (6.8) | 0.107 |
|  |  | **Frontal** | 37 | 15 (34.9) | 22 (50) | 0.154 |
|  |  | **Temporal** | 53 | 21 (47.7) | 32 (72.7) | **0.017** |
|  |  | **Central** | 14 | 7 (15.9) | 7 (15.9) | 1 |
|  | **Spike or sharp wave** | **Parietal** | 9 | 5 (11.4) | 4 (9.1) | 0.725 |
|  |  | **Occipital** | 5 | 5 (11.4) | - | **0.021** |
|  |  | **Frontal** | 33 | 19 (43.2) | 14 (32.6) | 0.307 |
|  |  | **Temporal** | 55 | 25 (56.8) | 30 (68.2) | 0.271 |
|  |  | **Central** | 9 | 3 (6.8) | 6 (13.6) | 0.291 |
| **Ictal EEG** | **Rhythmic teta/delta activity** | **Parietal** | 6 | 4 (9.1) | 2 (4.5) | 0.398 |
|  |  | **Occipital** | 1 | 1 (2.3) | - | 0.315 |
|  |  | **Frontal** | 47 | 28 (63.6) | 19 (43.2) | 0.054 |
|  |  | **Temporal** | 62 | 27 (61.4) | 35 (79.5) | 0.062 |
|  |  | **Central** | 12 | 6 (13.6) | 6 (13.6) | 1 |
|  | **Spike or sharp wave** | **Parietal** | 8 | 4 (9.1) | 4 (9.1) | 1 |
|  |  | **Occipital** | 5 | 4 (9.1) | 1 (2.3) | 0.167 |
|  |  | **Frontal** | 20 | 13 (29.5) | 7 (15.9) | 0.127 |
|  |  | **Temporal** | 24 | 14 (31.8) | 10 (22.7) | 0.338 |
|  |  | **Central** | 7 | 3 (6.8) | 4 (9.1) | 0.694 |
|  | **Rapid generalization** | | 35 | 19 (43.2) | 16 (36.4) | 0.332 |
